# Expression Patterns of Coagulation Factor XIII Subunit A on Leukemic Lymphoblasts Correlate with Clinical Outcome and Genetic Subtypes in Childhood B-cell Progenitor Acute Lymphoblastic Leukemia

**DOI:** 10.3390/cancers12082264

**Published:** 2020-08-13

**Authors:** Bettina Kárai, Katalin Gyurina, Anikó Ujfalusi, Łukasz Sędek, Gábor Barna, Pál Jáksó, Peter Svec, Eszter Szánthó, Attila Csaba Nagy, Judit Müller, Réka Simon, Ágnes Vojczek, István Szegedi, Lilla Györgyi Tiszlavicz, Jerzy R. Kowalczyk, Alexandra Kolenova, Gábor T. Kovács, Tomasz Szczepański, Michael Dworzak, Angela Schumich, Andishe Attarbaschi, Karin Nebral, Oskar A. Haas, János Kappelmayer, Zsuzsanna Hevessy, Csongor Kiss

**Affiliations:** 1 Department of Laboratory of Medicine, University of Debrecen, 4032 Debrecen, Hungary; karai.bettina@med.unideb.hu (B.K.); ujfalusi.aniko@med.unideb.hu (A.U.); szantho.eszter@med.unideb.hu (E.S.); kappelmayer@med.unideb.hu (J.K.); 2 Department of Pediatrics, University of Debrecen, 4032 Debrecen, Hungary; katalingyurina@gmail.com (K.G.); iszegedi@med.unideb.hu (I.S.); 3 Department of Microbiology and Immunology, Medical University of Silesia, 40-055 Katowice, Poland; lsedek@sum.edu.pl; 4 1st Department of Pathology and Experimental Cancer Research, Semmelweis University, 1085 Budapest, Hungary; barna.gabor@med.semmelweis-univ.hu; 5 Department of Pathology, Scientific University of Pécs, 7622 Pécs, Hungary; jakso.pal@pte.hu; 6 Department of Pediatric Hematology and Oncology, National Institute of Children’s Diseases and Comenius University Bratislava, 833 40 Bratislava, Slovakia; peter.svec@gmail.com (P.S.); sasa.kolenova@gmail.com (A.K.); 7 Department of Preventive Medicine, Faculty of Public Health, University of Debrecen, 4028 Debrecen, Hungary; nagy.attila@sph.unideb.hu; 8 2nd Department of Pediatrics, Semmelweis University, 1094 Budapest, Hungary; muller.judit@med.semmelweis-univ.hu (J.M.); kovacs.gabor1@med.semmelweis-univ.hu (G.T.K.); 9 Department of Pediatric Hematology-Oncology, BAZ county university hospital pediatric center, 3526 Miskolc, Hungary; reka.simondr@gmail.com; 10 Department of Pediatrics, Scientific University of Pécs, 7622 Pécs, Hungary; vojczeka@gmail.com; 11 Department of Pediatrics, Scientific University of Szeged, 6720 Szeged, Hungary; tiszlaviczlilla@yahoo.com; 12 Department of Pediatric Hematology, Oncology and Transplantology, Lublin Medical University, 20-059 Lublin, Poland; jerzy.kowalczyk@uszd.lublin.pl; 13 Department of Pediatric Hematology and Oncology, Medical University of Silesia Zabrze, 41-808 Zabrze, Poland; szczep57@poczta.onet.pl; 14 Children’s Cancer Research Institute and St. Anna Children’s Hospital, Pediatric Clinic, Medical University of Vienna, 1090 Vienna, Austria; michael.dworzak@stanna.at (M.D.); angela.schumich@ccri.at (A.S.); andishe.attarbaschi@stanna.at (A.A.); karin.nebral@labdia.at (K.N.); oskar.haas@labdia.at (O.A.H.)

**Keywords:** children, acute lymphoblastic leukemia, B-cell progenitor, Factor XIII subunit A, genetic risk categories, survival, middle-income countries

## Abstract

Background: Based on previous retrospective results, we investigated the association of coagulation FXIII subunit A (FXIII-A) expression pattern on survival and correlations with known prognostic factors of B-cell progenitor (BCP) childhood acute lymphoblastic leukemia (ALL) as a pilot study of the prospective multi-center BFM ALL-IC 2009 clinical trial. Methods: The study included four national centers (*n* = 408). Immunophenotyping by flow cytometry and cytogenetic analysis were performed by standard methods. Copy number alteration was studied in a subset of patients (*n* = 59). Survival rates were estimated by Kaplan-Meier analysis. Correlations between FXIII-A expression patterns and risk factors were investigated with Cox and logistic regression models. Results: Three different patterns of FXIII-A expression were observed: negative (<20%), dim (20–79%), and bright (≥80%). The FXIII-A dim expression group had significantly higher 5-year event-free survival (EFS) (93%) than the FXIII-A negative (70%) and FXIII-A bright (61%) groups. Distribution of intermediate genetic risk categories and the “B-other” genetic subgroup differed significantly between the FXIII-A positive and negative groups. Multivariate logistic regression confirmed independent association between the FXIII-A negative expression characteristics and the prevalence of intermediate genetic risk group. Conclusions: FXIII-A negativity is associated with dismal survival in children with BCP-ALL and is an indicator for the presence of unfavorable genetic alterations.

## 1. Introduction

Acute lymphoblastic leukemia (ALL) is the most frequent neoplastic disorder in children, which was virtually incurable before the 1960-ies. Nowadays, about 90% of children with ALL experience long-term survival in high-income countries using risk-tailored combined chemotherapy. ‘BFM ALL Intercontinental’ (ALLIC) is a network of national groups and single centers of the international Berlin-Frankfurt-Münster Study Group (iBFM-SG) with restricted resources. ALLIC members do not have a regular access to the most advanced molecular technologies required from participants of most recent ALL clinical trials [1,2,3]. Yet, the ALL IC-BFM 2002 clinical trial demonstrated successful co-operation of the participating groups resulting in competent event-free survival (EFS) and overall survival (OS) rates which exceeded historical results of the members [4]. Toxic mortality in ALL IC-BFM 2002 was higher than in parallel clinical trials of the Children’s Oncology Group (COG) and other BFM consortia. This fact highlights the need for the identification of potent biomarkers allowing precise stratification of risk groups of pediatric ALL. A broad spectrum of chromosomal abnormalities, submicroscopic structural genetic alterations and sequence mutations gained a role as prognostic and predictive biomarkers in childhood ALL, in addition to conventional risk factors, such as age, sex, initial white blood cell count (WBC), and response-to-treatment measures [5,6,7,8]. Cell surface and intracellular (cytoplasmic; cy) proteins of leukemic cells may provide additional prognostic biomarkers [9,10]. Identification of novel leukemia-associated protein biomarkers to be detected by flow cytometry (FC) is of special interest for ALLIC members [11]. FC is an economic, powerful, and user-friendly method. Recently, next generation flow cytometry was shown to reach the sensitivity and specificity of advanced molecular technologies in the detection of minimal residual disease (MRD) [12]. The consortium developed a multiparameter FC-based MRD detection method as a pilot project of ALL IC-BFM 2002 [13]. Result of mid-induction, day 15 bone marrow (BM) FC-MRD was incorporated in the risk assessment approach of the next ALLIC clinical trial, ALL IC-BFM 2009 (EuDract: 2010-019722-13, unpublished data) [14]. ALLIC FC laboratories became members of the International BFM Flow-network [8,15].

### Presence and Role of FXIII-A in Different Cell Types

FXIII is a pro-transglutaminase circulating in plasma as a tetramer of subunit A and B (FXIII-A_2_B_2_). FXIII-A but not FXIII-B is present physiologically as a dimer, FXIII-A_2_ in several types of cells, such as megakaryocytes, platelets, monocytes/macrophages, dendritic cells, mast cells, sebocytes, preadipocytes, osteoblasts, chondrocytes, and cornea cells. Excellent reviews summarized the functions of FXIII in health and disease [16,17,18]. In addition to normal cells, FXIII-A is present in transformed cells of benign and malignant neoplastic conditions, such as hamartomas of tuberous sclerosis, juvenile xanthogranuloma, dermatofibroma, other fibrovascular tumors, anaplastic hemangiopericytoma, clear cell carcinomas (CCC), biphenotypic sinonasal sarcoma, myeloid leukemias of the monoblast/monocyte and megakaryocyte lineages, and acute promyelocytic leukemia (APL) [19,20,21,22,23,24,25,26,27]. Our group was the first to identify leukemic lymphoblasts of patients with BCP-ALL as a previously unknown expression site for FXIII-A. FXIII-A expression was only observed in B-cell progenitor (BCP) lymphoblasts and neither in mature normal and leukemic B-cells, nor in normal BM B-cell precursors. Immunophenotypic markers of FXIII-A positive BCP lymphoblasts did not differ from FXIII-A negative BCP lymphoblasts [28].

In contrast to the role of FXIII in circulation, little is known about the function of the FXIII-A_2_. The role of FXIII-A_2_ was demonstrated in cytoskeletal remodeling and in the complex processes of phagocytosis, chemotaxis and cell adhesion. Translocation of FXIII-A_2_ from the cytoplasm to the nucleus was demonstrated, where the enzyme may react with as yet unidentified substrates [16,18,29]. FXIII-A_2_ expression in CCC cells was implicated in the pathogenesis of thromboembolic complications [25]. In neoplastic fibroblasts preferentially expressing FXIII-A_2_ as compared with normal fibroblasts, FXIII-A_2_ was suggested to support cell proliferation [23,30].

Previously, our group described that FXIII-A was expressed in leukemic BCP lymphoblasts in addition to known expression sites. In a retrospective single-center cohort of children with ALL treated with the ALL IC-BFM 2002 protocol, expression of FXIII-A was correlated with statistically significant survival advantage [31]. According to the observations of on one of our further study, FXIII-A expression intensity at the protein level seemed an important marker to stratify better the BCP-ALL. Using oligonucleotide microarray analysis and RT-Q-PCR method, we have also demonstrated that the intensity of expression of the FXIII-A_1_ gene (*F13A1*) at the mRNA level correlated with the three characteristic FXIII-A protein expression groups defined by FC, i.e., FXIII-A-negative, -dim, and –bright. The three different FXIII-A expression groups, i.e., FXIII-A-negative, -dim, and –bright, defined three distinct gene expression signatures. The differentially expressed genes had biologically and clinically relevant functions and were associated with leukemia and other forms of cancer. Moreover, gene expression profile of FXIII-A-negative samples exhibited an almost complete overlap with that of samples classified as belonging to the ‘B-other’ genetic category. These data suggested that the three different FXIII-A expression profiles defined by FC might characterize novel subgroups of pediatric BCP-ALL [32]. Similar associations between expression intensity of biomarkers both at the mRNA and protein levels and clinical relevance have been observed to exist in T-ALL and in AML [33,34].

Our aim was to investigate the association of FXIII-A expression pattern on survival figures and correlations with known clinical and genetic prognostic factors in a multi-center study within the frames of the prospective ALL IC-BFM 2009 clinical trial. We also wanted to identify cases characterized by adverse risk so that these can be more closely monitored not only by widely used methods but also the examination of FXIII-A pattern.

## 2. Results

### 2.1. Clinical Significance of Different Expression Patterns of FXIII-A by B-Cell Progenitor (BCP) Lymphoblasts

**Figure 1:**
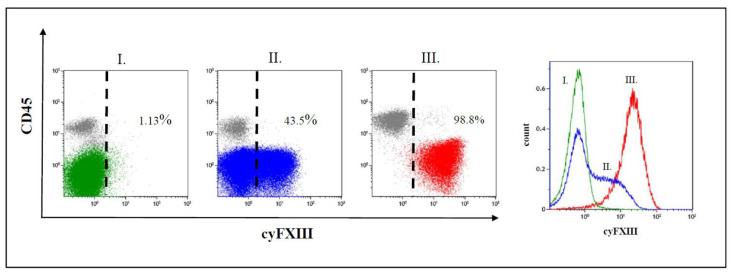
Representative dot plots and histograms of leukemic lymphoblasts. There are three different patterns of cytoplasmic FXIII-A expression in terms of positivity of leukemic lymphoblasts (CD45 negative population): (**I**) negative expression pattern below 20%, (**II**) dim positive expression pattern between 20% and 79%, and (**III**) bright positive expression pattern ≥ 80% of leukemic lymphoblasts with an FXIII-A staining exceeding the intensity of negative controls, i.e., residual normal lymphocytes (grey). Based on FXIII-A expression intensity of normal residual lymphocytes, the threshold of positivity is marked by the dashed line on the respective dot-plots. Percentages are referring to lymphoblasts. The intensity of the FXIII-A expression increased continuously, as the histogram of lymphoblasts with dim expression pattern shows (indicated by blue colour), which excludes the existence of a distinct FXIII-A negative (green colour) and a FXIII-A bright (red colour) subpopulation.

We observed three different staining patterns of FXIII-A expression in terms of positivity of leukemic lymphoblasts: a negative pattern, a dim pattern, and a bright pattern (Figure 1) [35]. In case of the negative expression pattern, leukemic lymphoblasts overlapped with residual normal lymphocytes. In bright cases, the leukemic blast cell population separated almost completely from residual normal lymphocytes. In case of dim samples, leukemic lymphoblasts appeared as a broad but homogenous group with a partial overlap with residual normal lymphocytes on FC dot-plots. The histogram analysis of FXIII-A dim expression cases showed that the leukemic cell population exhibited a single group with a continuously increasing fluorescence intensity and excluded the existence of two distinct subpopulations, i.e., a negative and a bright one with respect to FXIII-A expression. Of the analyzed 408 cases, there were 137 negative, 189 dim, and 82 FXIII-A bright samples.

**Figure 2:**
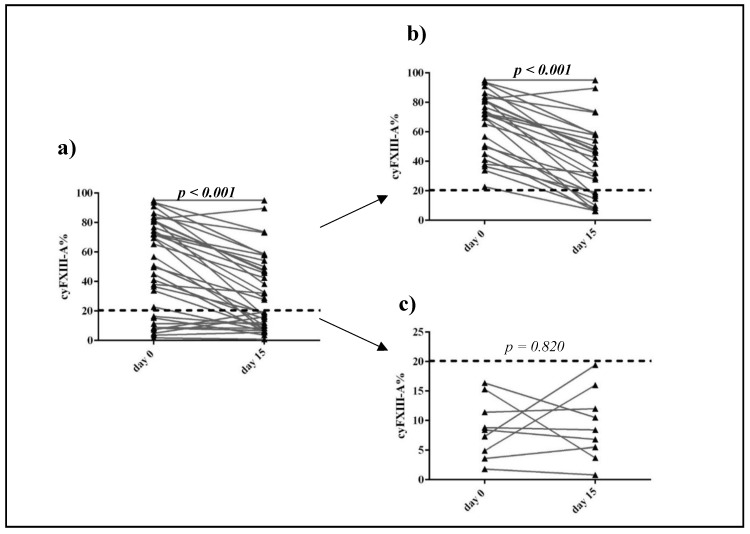
Ratio of FXIII-A expression of leukemic lymphoblast on Day 0 and Day 15. Thirty-six patients had FXIII-A expression data on Day 15 (**a**). Leukemic lymphoblasts were FXIII-A positive in 27 cases (**b**) and FXIII-A negative by 9 patients (**c**). FXIII-A expression of negative blasts was less than 20% (dashed line). *p* < 0.05 values were considered statistically significant, such *p* values are marked in bold.

In 36 patients, FXIII-A expression was evaluated in parallel at diagnosis and in the day 15 BM sample. We excluded patients of the Flow Low-risk (FLR) category because exact ratio of FXIII-A positive lymphoblasts below 0.1% could not be estimated due to the cytoplasmic expression of FXIII-A. The percentage of FXIII-A positive leukemic lymphoblasts of patients with Flow Medium- risk (FMR) and Flow High-risk (FHR) categories was significantly lower in Day 15 than in Day 0 samples (*p* < 0.001) [14]. FXIII-A expression of FXIII-A negative de novo cases did not change significantly by Day 15. Of the de novo FXIII-A negative cases, none surpassed the cut-off value of 20% for FXIII-A positivity by Day 15 (Figure 2).

**Figure 3:**
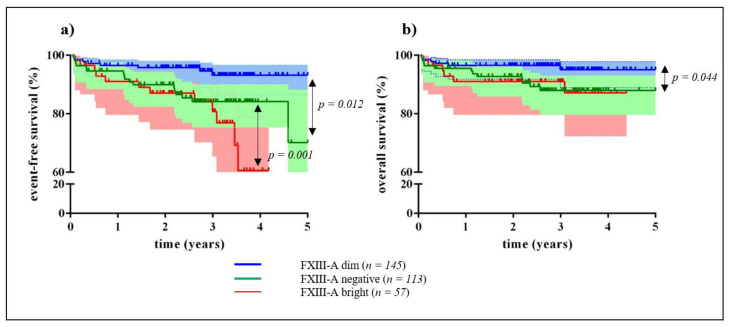
Event-free and overall survival of children with B-cell precursor acute lymphoblastic leukemia (ALL) by FXIII-A expression pattern. According to the Kaplan-Meier analysis, patients with FXIII-A dim lymphoblasts (blue line) had significantly higher 5-year event-free survival (EFS) (93%) compared with patients with FXIII-A bright lymphoblasts (red line; 61%) and with FXIII-A negative lymphoblasts (green line; 70%) (**a**). Mean follow-up time for the FXIII dim group was 1736 days (95% CI: 1675 and 1796 days); for the FXIII-A bright group 1277 days (95% CI: 1153 and 1400 days); and for the FXIII-A negative group 1588 days (95% CI: 1484 and 1692 days), with the last censored event at 31 days. Difference of 5-year OS was significant between patients with FXIII-A dim lymphoblasts (blue line; 95%) and patients with FXIII-A negative lymphoblasts (green line; 88%). Difference between the 5-year OS of the FXIII-A dim group and the FXIII-A bright group (red line; 87%) was not significant (**b**). Mean follow-up time for the FXIII-A dim group was 1755 days (95% CI: 1699 and 1810 days); for the FXIII-A bright group 1454 days (95% CI: 1341 and 1567 days); and for the FXIII-A negative group 1661 days (95% CI: 1572 and 1749 days), with the last censored event at 31 days. Statistically significant differences between the respective groups are marked with arrows (**a**,**b**). Shaded regions indicate that at any time point, there is a 95% chance that the interval contains the true percentage survival.

We investigated the association of FXIII-A expression pattern at diagnosis on the EFS and OS of patients using Kaplan-Meier survival analysis. EFS and OS of patients with FXIII-A positive BCP-ALL were not significantly different from that of patients with FXIII-A negative BCP-ALL (Figure S1). Investigating the three different FXIII-A expression patterns separately, a significant EFS advantage of the FXIII-A dim group was demonstrated when compared both with the FXIII-A negative (*p* = 0.012), and with the FXIII-A bright group (*p* = 0.001) (Figure 3a). The 5-year EFS of patients with dim, negative, and bright FXIII-A expression was 93%, 70%, and 61%, respectively. The 5-year OS of the FXIII-A dim group (95%) was significantly higher than that of the FXIII-A negative group (88%; *p* = 0.044). The difference between the 5-year OS of the FXIII-A dim and the FXIII-A bright group (87%) was not significant (Figure 3b).

The impact of categorical variables representing known risk factors on the two pairs of groups, i.e., the FXIII-A dim vs. FXIII-A negative groups and the FXIII-A dim vs. FXIII-A bright groups, was analyzed with the multivariable Cox regression model. Categorical variables, such as FXIII-A expression pattern, age, prednisone response, distribution of genetic risk categories, and distribution of the “B-other” genetic subgroup had significant effects on 5-year EFS and 5-year OS figures of the dim vs. negative FXIII-A groups. The effect of ALL BFM-IC 2009 risk groups was significant on the 5-year OS. In the multivariate analysis model, only genetic risk category (good vs. intermediate) remained significant (Table 1). Significant differences between survival figures of the dim vs. bright FXIII-A groups were seen in case of FXIII-A expression pattern, ALL BFM-IC 2009 risk categories (BFM-HR vs. BFM-IR) and the genetic risk categories. In the multivariate Cox analysis, FXIII-A expression pattern and genetic risk categories remained significant (Table 2). FXIII-A negative and bright group associated with adverse outcome. Comparing the two groups, the prednisone response and genetic risk categories differed significantly. Poor prednisone response was more frequent in FXIII-A negative group, while the presence of patients with high-risk genetic alterations was higher in FXIII-A bright group (Table 3).

Copy number alteration (CNA) was investigated in 59 patients, 20 with negative, 26 with dim, and 13 with bright FXIII-A expression pattern. Poor prognostic CNA were detected in a slightly higher ratio (7/20) in the negative FXIII-A group than in the dim (4/26), and the bright FXIII-A (1/13) groups (Table S2). The differences were not significant.

### 2.2. Correlation of FXIII-A Expression Patterns with Conventional Clinical, Minimal Residual Disease (MRD), and Genetic Risk Factors

Relationships between the FXIII-A expression patterns and other known risk factors were investigated with Pearson’s Chi square test and multinomial logistic regression models. The different FXIII-A expression patterns did not correlate significantly with either risk stratification according to ALL BFM-IC 2009 or FC MRD risk categories. The intermediate genetic risk category was significantly more prevalent in the FXIII-A negative group than in the FXIII-A dim or bright group (*p* = 0.009 and *p* = 0.039, respectively). Correlation between negative and positive FXIII-A expression patterns and the “B-other” subgroup was equally strong (*p* = 0.008 FXIII-A negative and dim group; *p* = 0.004 FXIII-A negative and bright group).

Patients with FXIII-A dim or bright lymphoblasts had lower chance for having “B-other” alteration (OR: 0.49 and 0.36, respectively). This association persisted after adjusting for categorical variables, as sex, age, and WBC (Table 4). iAMP21 was also significantly more (*p* = 0.029) prevalent in the FXIII-A negative group as compared with the two other FXIII-A expression groups. Other recurrent genetic aberrations showed a similar distribution within the three FXIII-A expression groups.

## 3. Discussion

In this study we investigated the potential usefulness of FXIII-A as a risk-associated biomarker in BCP-ALL of children treated within the frames of the ALL BFM-IC 2009 clinical trial. In a previous small-scale, single-center, retrospective study we observed a survival advantage of children with FXIII-A positive (≥20%) vs. FXIII-A negative BCP-ALL treated with the ALL BFM-IC 2002 protocol [31]. The strength of the present study was that four national groups, belonging to two different childhood ALL treatment consortia (Polish, Hungarian, Slovak, and Austrian) investigated FXIII-A expression by quality-controlled multiparameter FC and delivered clinically relevant data of patients participating in this multi-center, prospective clinical trial. The high number of cases included in this study enabled the more detailed examination of FXIII-A expression patterns.

In a recent publication, our group investigated the gene expression profile of the three different expression types of FXIII-A in BCP-ALL. The three groups had unique gene expression signatures according to FXIII-A expression pattern. The gene expression pattern of the FXIII-A negative subgroup overlapped with the gene expression profile of the “B-other” genetic subgroup [32].

The effect of FXIII-A expression in the leukemic cell population on survival was studied in patients with APL and conflicting results were reported [29,36]. The two groups applied different methods, i.e., FC and immunohistochemistry to detect manifestation of FXIII-A in leukemic promyelocytes. However, neither of the two groups made a distinction between bright and dim expression pattern of FXIII-A in APL cells, only patients with FXIII-A positive and negative expression patterns were compared which might explain the opposite conclusions of the two investigations.

Most children with BCP-ALL displayed a dim FXIII-A expression. This subpopulation had a superior outcome over both the bright and the negative FXIII-A groups according to the univariate Kaplan-Meier analysis. The inferior survival chances of the FXIII-A negative subgroup were explained by the significantly higher prevalence of patients with intermediate genetic risk group, and, within this group, patients belonging to the “B-other” category, compared with the FXIII-A positive group. Patients with “B-other” BCP-ALL were shown to have a higher risk for relapse [7,32]. We also demonstrated the significant prognostic value of the “B-other” genetic category on EFS and OS of patients with negative vs. dim FXIII-A expression by the multivariable Cox regression analysis [37]. We observed a trend of association of poor prognostic CNAs with FXIII-A negativity; however, probably because of the small sample size of the multiplex ligation dependent probe amplification (MLPA) investigation, the difference was not statistically significant.

Comparing the expression of FXIII-A of leukemic lymphoblasts on Day 0 and Day 15, a significant decrease was detected in the ratio of FXIII-A positive lymphoblasts of BM samples by day 15, while FXIII-A expression did not appear on originally FXIII-A negative lymphoblasts. Complemented with the results of ES and OS, this finding suggested a preferential clearance of FXIII-A positive blast cells over negative ones.

Multivariate Cox analysis showed significant impacts of the presence of high-risk and intermediate-risk genetic alterations on survival figures of patients with bright FXIII-A expression as compared with that of the dim FXIII-A group. In the univariate Cox regression analysis there was a significant association between the dismal EFS and OS of the bright FXIII-A expression group and the BFM-HR category. These results suggest that unfavorable genetic alterations may supersede the beneficial effect of FXIII-A expression in children with BCP-ALL.

In a previous study, results suggested that not even the lowest possible MRD cut-off was associated with the best overall survival for all patients [38]. Therefore, novel markers, such as FXIII-A expression pattern may gain importance in achieving optimal stratification of patients. By improving risk stratification, the new biomarkers may contribute to developing a more refined risk-adjusted, or even personalized treatment.

There were several limitations of this study. Of note, survival rates of the present study do not represent either the total ALL BFM-IC 2009 study population, or any of the patient subpopulations of the participating national groups. First, only children with BCP-ALL were investigated. Second, FXIII-A labeling and measurement was performed on the basis of the availability of the anti-FXIII-A monoclonal antibody. Third, patients of the Polish group were not randomized. Fourth, a substantial number of patients were enrolled within the last four years in the study which may alter ultimate 5-year EFS and OS figures. Yet, follow-up times were sufficient to demonstrate statistically significant differences between groups with different FXIII-A expression patterns. A complete genetic diagnosis was available only from 214/317 patients and copy number alterations by MLPA was investigated on a small subset of patients.

## 4. Materials and Methods

### 4.1. Study Cohort

Diagnostic BM samples and clinical data were collected from 408 children with BCP-ALL treated by the Polish (188), Hungarian (114), and Slovak (13) groups of the ALL IC consortium and by the Austrian (93) group of the AIEOP-BFM consortium between 2011 and 2018. Patients with Down-syndrome, t(9;22), and infants were excluded. Patients were unselected for FXIII-A labeling. In a subset of 36 children treated at the Debrecen Center (Hungary), diagnostic and day 15 BM samples were studied in parallel for FXIII-A labeling. Of the 408 patients 310 had a complete genetic test. Results of these patients were included in the regression analyses. Of these patients, six patients in Hungary, eight patients in Poland, and two patients in Slovakia underwent allogenic haematopoietic stem cell transplantation. In a subset of 59 children treated at the Debrecen and Budapest Centers (Hungary), multiplex ligation dependent probe amplification (MLPA) assay was performed to investigate copy number alterations (CNA).

Polish, Hungarian, and Slovak patients were treated according to ALL IC-BFM 2009 protocol, while Austrian patients were treated according to AIEOP-BFM 2009. Due to the difference in the applied treatment protocols, the data from the Vienna cases were used only in the analysis of the association between the initial variables and FXIII-A expression; the association of FXIII-A expression on survival was examined exclusively by comparing data of patients treated with the same protocol. Among the ALLIC cohort of patients, i.e., patients from Hungary, Poland, and Slovakia there were 21 relapses. Relapsed ALL patients were treated according to ALL-REZ BFM 2002 protocol/clinical trial (ClinicalTrials.gov identifier:NCT00114348) [39,40]. Risk estimation at diagnosis and evaluation of prednisone response in day 8 peripheral blood (PB) samples were retained from ALL IC-BFM 2002 [4]. Low hypodiploidy (chromosome number <45), and iAMP21 were considered as high-risk (BFM-HR) features in ALL IC-BFM 2009 in addition to ALL IC-BFM 2002. Evaluation of mid-induction (day 15) BM samples was based on FC-MRD analysis. An MRD load by FC <0.1% was considered as FLR, FC-MRD between 0.1% and <10% was considered as FMR, and FC-MRD ≥10% was considered as FHR. Only patients stratified into the standard-risk (BFM-SR) group according to conventional risk factors and having FLR status remained in the BFM-SR group. Patients with FMR and FHR status were upgraded into the intermediate-risk (BFM-IR) group, and into the BFM-HR group, respectively. Patients of the BFM-IR group based on conventional risk factors but displaying an FHR status, were upgraded into the BFM-HR group. ALL BFM-IC 2009 accepted a risk estimation based on morphologic evaluation of day 15 BM samples in the lack of an FC-MRD analysis. However, FC-MRD analysis was performed in every case in the present study.

Standard treatment arms of ALL IC-BFM 2009 were identical with that of ALL IC-BFM 2002 [4]. Patients in Hungarian and Slovak centers were randomized. Patients with BFM-IR and BFM-HR disease received either standard early intensification as in ALL BFM-IC 2002, or an “augmented” early intensification, applied previously by BFM and Children’s Cancer (CCG) groups [4,41,42]. Patients with IR BCP-ALL were randomized to receive either 2 g/m^2^ methotrexate (Mtx) as in ALL BFM-IC 2002 or 5 g/m^2^ Mtx, as in ALL-BFM 86 in consolidation [43]. The Polish group did not perform randomization: patients of the BFM-IR group received standard and patients of the BFM-HR group received augmented early intensification. Patients with IR BCP-ALL received 2 g/m^2^ Mtx. Initial clinical characterization and risk stratification of study patients are shown in Table S1.

The study was approved by the Scientific Research Ethical Committee of the Medical Research Council of Hungary (no 43033-1/2014/EUK(423/2014)) and was performed according to the 2008 Declaration of Helsinki. Written informed consent was obtained from legal guardians of participating patients.

### 4.2. Immunophenotype Analysis

**Figure 4:**
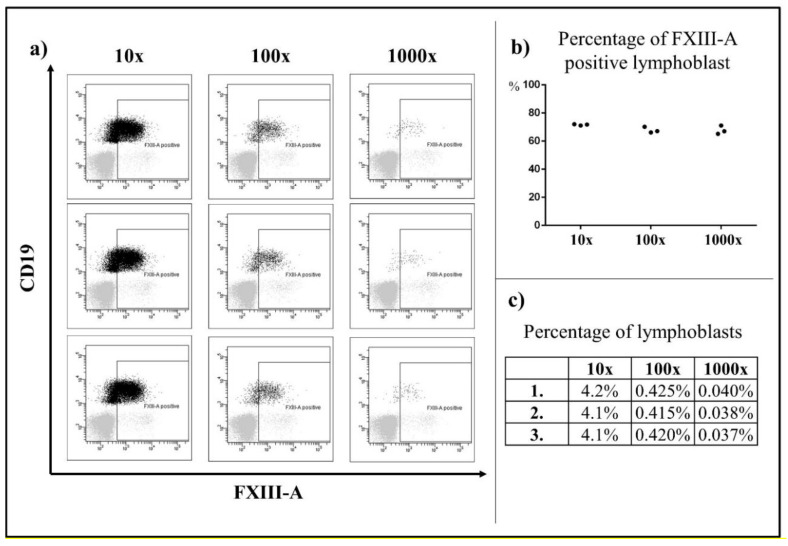
Detection of FXIII-A expression on analysis of serial dilutions. Based on a three parallel serial dilutions (10×; 100×; 1000×), the percentage of FXIII-A expression in residual lymphoblasts could be clearly assessed when the percentage of lymphoblasts was above 0.04%. Black color indicates the residual lymphoblasts, grey color represents the mature lymphocytes, which were the negative control. (**a**) The percentages of FXIII-A positive lymphoblasts within all lymphoblasts were similar in the three parallel staining instances (**b**) The percentages of lymphoblasts served as the internal control of the serial dilutions (**c**).

FC investigations were performed as described [13,28,31]. Briefly, samples were analyzed by 5–8 color labeling procedure using FacsCantoII (Becton Dickinson, Franklin Lakes, NJ, USA) and Navios and FC-500 (Beckman Coulter, Brea, CA, USA) flow cytometers. Lineage assignment was determined according to EGIL criteria [44]. Surface and ic staining was performed according to standard protocols, with the ALLIC-2009 suggested panel of monoclonal antibodies. Generation and fluorescent isotihocyanate (FITC) labeling of mouse monoclonal antibody against FXIII-A was carried out as previously described [45]. To examine the sensitivity of FXIII-A determination, the percentage of FXIII-A expression in residual lymphoblasts was analyzed in three parallel serial dilutions (10×, 100×, 1000×). The percentage of FXIII-A expression in residual lymphoblasts could be clearly assessed when the percentage of lymphoblasts was above 0.04% (Figure 4).

Each center used the same tube to assess FXIII-A labeling: FXIII-A (FITC)–CD10(PE)–CD45(PerCP-Cy5.5)–CD19(APC). The threshold of positivity was set to 20% positive leukemic cells for a certain immunophenotype marker. To examine the significance of the FXIII-A expression in more detail, three groups were defined: BCP-ALL with FXIII-A negative blasts (<20% FXIII-A positive lymphoblasts; FXIII-A negative group), BCP-ALL with FXIIII-A dim positive expression (20–79% FXIII-A positive lymphoblasts; FXIII-A dim group), and BCP-ALL with FXIII-A bright positive expression (≥80% FXIII-A positive lymphoblasts; FXIII-A bright group). To determine FXIII-A expression of leukemic cells, normal residual lymphocytes served as a negative control [19,28]. FC data were analyzed by FACSDiva (Becton Dickinson, Franklin Lakes, NJ, USA) or Kaluza (Beckman Coulter, Brea, CA, USA) software. Flow cytometers were controlled applying Cytometer Setup&Tracking (Becton Dickinson, Franklin Lakes, NJ, USA) or Flow Check Pro (Beckman Coulter, Brea, CA, USA) fluorescent microbeads. All laboratories participated in the UK-NEQAS Leukocyte Immunophenotyping MRD program and in the ALLIC Annual Ring Trials successfully.

### 4.3. Genetic Investigations

The unstimulated 24 h cultures of BM were subjected to cytogenetic analysis using standard protocol. Subsequently fluorescence in situ hybridization (FISH) was performed on cells from the same BM samples according to manufacturer’s recommendations using commercially available probe sets (*BCR-ABL, ETV6-RUNX1, MLL)* (MetaSystems, Altlussheim, Germany). The low-risk group was formed of patients with t(12;21)/*ETV6/RUNX1* or high hyperdiploidy (51–65 chromosome), while the high-risk group was made up of patients with *MLL* translocations, iAMP21, complex karyotype, near haploidy (chromosome number 23–29), and low hypodiploidy (chromosome <45). Finally, an intermediate-risk group was established from patients with t(1;19) and all other genetic subgroups not fitting in the low- and high-risk categories, including the “B-other” subgroup [5,6].

Genomic DNA was extracted from BM samples using QIAamp DNA Blood Mini Kit (QIAGEN, Hilden, Germany). SALSA MLPA P335-B2 ALL-IKZF1 probe mix was used to perform MLPA analysis (MRC-Holland, Amsterdam, The Netherlands). The kit includes probes for *IKZF1, CDKN2A/B, PAX5, EBF1, ETV6, BTG1, RB1* genes and the *PAR1* region (*CRLF 2, CSF2RA, IL3RA*). No deletion of *IKZF1, CDKN2A/B, PAR1, BTG1, EBF1, PAX5, ETV6*, or *RB1*, isolated deletions of *ETV6, PAX5,* or *BTG1, ETV6* deletions with a single additional deletion of *BTG1, PAX5*, or *CDKN2A/B* were considered as good; single deletions of *CDKN2A/B*, and combined deletions of *CDKN2A/B/PAX5* were considered as intermediate; and any deletion of *IKZF1, PAR1, EBF1*, or *RB1,* and all other CNA profiles not mentioned above were considered as poor prognostic factors according to CNA [46,47,48,49].

### 4.4. Statistical Analysis

Normal distribution was tested by the Shapiro-Wilk test. To compare two groups, we used paired Student’s t-test for parametric and Wilcoxon test for non-parametric data. Where there were more than two groups, data were analyzed by the Kruskal-Wallis test. Dunn’s multiple comparison test was applied as post hoc test. The initial parameters of patients, such as age and WBC count, were transformed to categorical variables on the basis of their prognostic impact.

Dichotomous categorical variables were compared using Pearson’s Chi square test and multinomial logistic regression to analyse multiple variables. *p*-values <0.05 were considered as significant. Survival analysis was carried out by the Kaplan-Meier survival estimator. Hazard ratios (HR) and 95% confidence intervals (CI) were estimated by Cox proportional hazard model analysis. The end point of event-free analysis was defined as the time elapsed between the diagnosis of ALL and the first relapse or death. Statistical analysis and the creation of figures were performed using STATA/IC 14.2 (College Station, TX, USA), SPSS 20.0 (Chicago, IL, USA) and GraphPad Prism 6.0 (San Diego, CA, USA) statistical programs.

## 5. Conclusions

The intracellular marker, FXIII-A has three different expression patterns in leukemic BCP lymphoblasts as determined by FC, i.e., FXIII-A bright positive, dim positive and FXIII-A negative expression. Our previous study showed that the three different staining patterns were associated with three distinct gene expression signatures. In the present study we demonstrated statistically significant and clinically meaningful differences in children with BCP-ALL according to FXIII-A expression pattern by FC which can be incorporated in the risk estimation strategy of a new ALL BFM-IC clinical trial. FXIII-A expression status of leukemic lymphoblasts can be easily and accurately determined by FC. FXIII-A negative expression pattern was a prognostic indicator of dismal survival in children with BCP-ALL due to the significant association between the FXIII-A negative expression pattern and the “B-other” genetic category. Similar associations between expression intensity of biomarkers both at the mRNA and protein levels and clinical relevance have been observed to exist in T-ALL and in AML. Patients with FXIII-A negative BCP lymphoblasts should be extensively investigated with sophisticated genetic and molecular methods so as to provide these patients an optimal risk-adjusted treatment.

## Figures and Tables

**Table 1:** Association of categorical variables on event-free survival (EFS) and OS of the FXIII-A negative vs. dim groups by univariate and multivariate Cox regression analysis

Variables	Event-Free Survival			Overall Survival		
	HR	95% CI	*p*-Value	HR	95% CI	*p*-Value
	**Univariate Analysis**					
FXIII-A expression pattern						
FXIII-A negative	2.91	1.17–7.21	**0.021**	2.63	0.88–7.84	0.084
FXIII-A dim	0.34	0.14–0.85		0.38	0.13–1.14	
Age						
1–5 years	0.39	0.17–0.92	**0.032**	0.24	0.08–0.71	**0.010**
≥6 years	2.56	1.10–6.05		4.20	1.41–12.56	
WBC						
<20,000	0.69	0.29–1.66	0.407	0.60	0.21–1.71	0.334
≥20,000	1.45	0.60–3.51		1.69	0.58–4.86	
Sex						
Male	1.53	0.62–3.79	0.360	1.87	0.59–6.00	0.291
Female	0.65	0.26–1.62		0.54	0.17–1.71	
prednisone response						
PGR	0.27	0.09–0.73	**0.010**	0.15	0.05–0.45	**0.001**
PPR	3.75	1.37–10.24		6.71	2.25–20.03	
FC risk categories						
FLR/FMR	1.43	0.40–5.13	0.581	1.18	0.25–5.54	0.838
FLR/FHR	3.37	0.78–14.58	0.104	3.40	0.62–18.56	0.158
ALLIC BFM 2009 classification						
BFM-HR/BFM-SR	0.12	0.01–0.95	**0.044**	0.00		
BFM-HR/BFM-IR	0.41	0.16–1.05	0.063	0.32	0.11–0.92	**0.035**
Genetic risk categories						
good/intermediate	7.12	2.07–24.73	**0.002**	13.8	1.77–107.9	**0.012**
good/high	23.7	4.72–119.3	**<0.001**	63.1	6.50–613.3	**<0.001**
B-other						
Recurrent genetic abnormalities	0.26	0.10–0.68	**0.006**	0.27	0.08–0.86	**0.027**
B-other	3.82	1.48–9.86		3.71	1.16–11.85	
	**Multivariate Analysis**					
FXIII negative	2.29	0.85–6.20	0.102	1.70	0.51–5.71	0.390
Genetic risk categories						
good/intermediate	5.78	1.63–20.52	**0.007**	10.0	1.24–81.4	**0.031**
good/high	17.4	2.88–104.8	**0.002**	30.5	2.63–353.4	**0.006**

*p* < 0.05 values were considered statistically significant, such *p* values are marked in bold.

**Table 2:** Association of categorical variables on event-free survival (EFS) and OS of the FXIII-A dim vs. bright groups by univariate and multivariate Cox regression analysis.

Variables	Event-Free Survival			Overall Survival		
	HR	95% CI	*p*-Value	HR	95% CI	*p*-Value
	**Univariate Analysis**					
FXIII-A expression pattern						
FXIII-A dim	0.24	0.09–0.64	**0.004**	0.32	0.09–1.04	0.057
FXIII-A bright	4.10	1.56–10.78		3.17	0.97–10.38	
Age						
1–5 years	0.52	0.19–1.37	0.186	0.32	0.10–1.05	0.060
≥6 years	1.92	0.73–5.06		3.13	0.96–10.28	
WBC						
<20,000	0.67	0.26–1.76	0.417	0.56	0.17–1.84	0.341
≥20,000	1.49	0.57–3.92		1.78	0.54–5.83	
Sex						
Male	1.43	0.53–3.86	0.483	2.08	0.55–7.86	0.278
Female	0.70	0.26–1.90		0.48	0.18–1.81	
prednisone response						
PGR	0.34	0.08–1.47	0.146	0.43	0.06–3.41	0.427
PPR	1.43	0.53–3.86		2.08	0.55–7.86	
FC risk categories						
FLR/FMR	0.90	0.27–2.83	0.857	1.33	0.24–5.54	0.863
FLR/FHR	1.25	0.23–6.81	0.801	2,59	0.36–18.43	0.342
ALLIC BFM 2009 classification						
BFM-HR/BFM-SR	0.39	0.098–1.59	0.192	0.17	0.02–1.49	0.110
BFM-HR/BFM-IR	0.27	0.09–0.78	**0.015**	0.21	0.06–0.73	**0.014**
Genetic risk categories						
good/intermediate	2.08	0.75–5.74	0.051	3.68	0.92–14.73	**0.065**
good/high	4.86	0.99–23.76	0.157	9.65	1.59–58.42	**0.014**
B-other						
Recurrent genetic abnormalities	0.55	0.21–1.44	0.225	0.41	0.12–1.33	0.137
B-other	1.80	0.70–4.78		2.46	0.75–8.07	
	**Multivariate Analysis**					
FXIII-A bright	3.92	1.41–10.84	**0.009**	3.90	0.99–15.31	**0.051**
Genetic risk categories						
good/intermediate	2.69	0.84–8.55	0.095	6.07	1.17–31.65	**0.032**
good/high	3.56	0.70–18.03	0.124	7.67	1.21–48.64	**0.031**

*p* < 0.05 values were considered statistically significant, such *p* values are marked in bold.

**Table 3:** Association of categorical variables on event-free survival (EFS) and OS of the FXIII-A negative vs. bright groups by univariate and multivariate Cox regression analysis.

Variables	Event-Free Survival			Overall Survival		
	HR	95% CI	*p*-Value	HR	95% CI	*p*-Value
	**Univariate Analysis**					
FXIII-A expression pattern						
FXIII-A negative	0.66	0.29–1.51	0.323	0.80	0.28–2.24	0.665
FXIII-A bright	1.52	0.66–3.47		1.26	0.45–3.54	
Age						
1–5 years	0.52	0.24–1.17	0.116	0.36	0.13–1.02	0.056
≥6 years	1.90	0.85–4.24		2.75	0.98–7.72	
WBC						
<20,000	0.49	0.21–1.13	0.093	0.68	0.23–1.98	0.477
≥ 20,000	2.06	0.89–4.76		1.48	0.50–4.32	
Sex						
Male	1.21	0.53–2.78	0.647	1.43	0.49–4.17	0.518
Female	0.82	0.36–1.89		0.70	0.24–2.05	
prednisone response						
PGR	0.36	0.13–0.96	**0.041**	0.28	0.09–0.87	**0.028**
PPR	2.81	1.04–7.58		3.61	1.15–11.35	
FC risk categories						
FLR/FMR	2.48	0.72–8.56	0.150	1.90	0.41–8.80	0.411
FLR/FHR	3.29	0.77–13.9	0.106	3.53	0.65–19.28	0.145
ALLIC BFM 2009 classification						
BFM-HR/BFM-SR	0.21	0.05–0.97	**0.045**	0.15	0.02–1.22	0.076
BFM-HR/BFM-IR	0.47	0.20–1.11	0.085	0.37	0.13–1.04	0.060
Genetic risk categories						
good/intermediate	2.14	0.89–5.17	0.091	2.50	0.75–8.30	0.135
good/high	6.48	1.69–24.84	**0.006**	10.5	2.33–47.2	**0.002**
B-other						
Recurrent genetic abnormalities	0.61	0.27–1.36	0.228	0.66	0.24–1.81	0.413
B-other	1.64	0.73–3.67		1.53	0.55–4.21	
	**Multivariate Analysis**					
FXIII-A bright	0.54	0.22–1.34	0.108	0.59	0.19–1.88	0.3751
Genetic risk categories						
good/intermediate	2.33	0.87–6.22	0.091	2.63	0.68–10.25	0.164
good/high	4.55	1.14–18.15	**0.032**	7.44	1.54–36.00	**0.013**

*p* < 0.05 values were considered statistically significant, such *p* values are marked in bold.

**Table 4:** Correlation of FXIII-A expression patterns with conventional clinical and “B-other” subgroup by multinomial logistic regression analysis.

FXIII-A Expression Patterns	*p* Value	OR	95% CI	
FXIII-A Negative	Reference Group (“Base Outcome”)			
FXIII-A dim				
“B-other” subgroup	**0.009**	**0.49**	**0.28**	**0.84**
Sex	**0.851**	**1.05**	**0.61**	**1.81**
Age	**0.197**	**0.69**	**0.39**	**1.21**
WBC	**0.093**	**1.69**	**0.92**	**3.10**
FXIII-A bright				
“B-other” subgroup	**0.004**	**0.35**	**0.18**	**0.71**
Sex	**0.726**	**0.89**	**0.46**	**1.72**
Age	**0.581**	**0.82**	**0.41**	**1.64**
WBC	**0.393**	**1.38**	**0.66**	**2.92**

*p* < 0.05 values were considered statistically significant, such *p* values are marked in bold.

## Supplementary Materials

The following are available online at https://www.mdpi.com/2072-6694/12/8/2264/s1, Figure S1: Event-free and overall survival of children with B-cell precursor acute lymphoblastic leukemia (ALL) of FXIII-A positive and negative groups, Table S1: Initial clinical characterization and risk stratification of study patients, Table S2: Distribution of copy number alterations (CNA) according to FXIII-A expression patterns.

## Abbreviations

FXIII-A	coagulation factor XIII subunit A
BCP	B-cell progenitor
ALL	acute lymphoblastic leukemia
BFM ALL-IC 2009	Berlin-Frankfurt-Münster inter-Continental 2009 clinical trial
iBFM-SG	international Berlin-Frankfurt-Münster Study Group
EFS	event-free survival
OS	overall survival
COG	Children’s Oncology Group
WBC	white blood cell count
ic	Intracellular
FC	flow cytometry
MRD	minimal residual disease
BM	bone marrow
MLPA	multiplex ligation dependent probe amplification
CNA	copy number alterations
AIEOP-BFM 2009	International Collaborative Treatment Protocol For Children And Adolescents With Acute Lymphoblastic Leukemia clinical trial
cyFXIII-A	cytoplasmic coagulation factor XIII subunit A
PB	peripheral blood
HR	high-risk
IR	intermediate risk
SR	standard risk
FLR	flow low-risk
FMR	flow medium-risk
FHR	flow high-risk
BFM-HR	BFM-high risk
BFM-SR	BFM standrard risk
BFM-IR	BFM intermediate risk
CCG	Children’s cancer group
Mtx	Methotrexate
FITC	fluorescent isotihocyanate
FISH	fluorescence in situ hybridization
CI	cinfidence intervals
HR	Hazard-ratio
CCC	clear cell carcinomas
APL	acute promyelocytic leukemia
PGR	prednisone good response
PPR	prednisone poor response
OR	Odds ratio
NA	not available
N	Number
