# Supplementary material for: Expression Patterns of Coagulation Factor XIII Subunit A on Leukemic Lymphoblasts Correlate with Clinical Outcome and Genetic Subtypes in Childhood B-cell Progenitor Acute Lymphoblastic Leukemia
Source: Cancers (Basel). 2020 Aug 13;12(8):2264. doi: 10.3390/cancers12082264 (PMC7463512; doi:10.3390/cancers12082264)
Supplement: Supplementary file 1 [file cancers-12-02264-s001.pdf]

cancers

MDPI

Expression Patterns of Coagulation Factor XIII Subunit A on Leukemic Lymphoblasts Correlate with Clinical Outcome and Genetic Subtypes in Childhood B-cell Progenitor Acute Lymphoblastic Leukemia

Bettina Kárai, Katalin Gyurina, Anikó Ujfalusi, Łukasz Sędek, Gábor Barna, Pál Jáksó, Peter Svec, Eszter Szánthó, Attila Csaba Nagy, Judit Müller, Réka Simon, Ágnes Vojczek, István Szegedi, Lilla Györgyi Tiszlavicz, Jerzy R. Kowalczyk, Alexandra Kolenova, Gábor T. Kovács, Tomasz Szczepański[3], Michael Dworzak, Angela Schumich, Andishe Attarbaschi, Karin Nebral, Oskar A. Haas, János Kappelmayer, Zsuzsanna Hevessy and Csongor Kiss

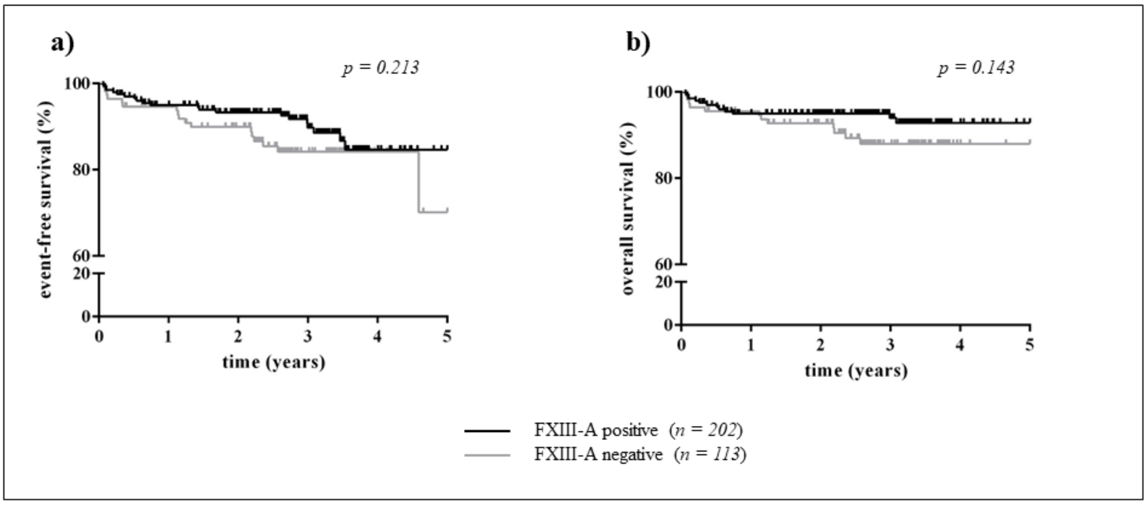

Figure S1. Event-free and overall survival of children with B-cell precursor acute lymphoblastic leukemia (ALL) of FXIII-A positive and negative groups.

Table S1. Initial clinical characterization and risk stratification of study patients.

Variables	*n*	%
Gender		
Male	216	52.9
Female	192	47.1
Age		
<6 years	283	69.4
≥6 years	125	30.6
WBC count		
<20,000 cells/µl	296	72.5
≥20,000 cells/µl	111	27.2
NA	1	0.3
FXIII-A expression ratio		
Negative (<20%)	137	33.6
Positive	271	66.4
dim (20–79%)	189	46.3
bright (≥80%)	82	20.1
Genetic risk categories *		
Good	184	45.1

Intermediate	118	28.9
Poor	17	4.2
NA	89	21.8
"B-other" genetic risk category		
Yes	110	26.9
No	209	51.2
NA	89	21.8
Prednisone response on day 8		
Good	378	92.6
Poor	24	5.9
NA	6	1.5
FC-MRD (day15)		
FLR: <0.1%	96	23.5
FMR: 0.1–10%	262	64.2
FHR: ≥10%	47	11.5
NA	3	0.7
clinical risk groups		
Standard risk	87	21.3
Intermediate risk	241	69.1
High risk	74	18.1
NA	6	1.5
Event-free survival		
Alive	361	88.5
Died/relapsed	47	11.5
Overall survival		
Alive	382	93.6
Died	26	6.4

* according to cytogenetics and FISH (see text). Abbreviations: n = number, WBC = white blood cell count, NA = not available, FC-MRD = minimal residual disease by flow cytometry, FLR = flow low risk, FMR = flow medium risk, FHR = flow high risk.

Table S2. Distribution of copy number alterations (CNA) according to FXIII-A expression patterns.

**FXIII-A Expression Pattern**	**Risk Estimation According to CAN ***			
	**Good**	**Intermediate**	**Poor**	**No CNA**
Negative	3	1	7	9
Dim	6	4	3	12
Bright	3	1	2	8

* See text.

© 2020 by the authors. Licensee MDPI, Basel, Switzerland. This article is an open access article distributed under the terms and conditions of the Creative Commons Attribution (CC BY) license (http://creativecommons.org/licenses/by/4.0/).
